# Comparative Assessment of the Nutritional, Phenolic, and Amino Acid Profiles, INFOGEST-Based Gastrointestinal Bioaccessibility, and Biological Activities of *Althaea officinalis*, *Jasminum officinale*, and *Viola odorata*

**DOI:** 10.3390/foods15193561

**Published:** 2026-10-08

**Authors:** Zehra Yaman, Kubra Feyza Erol, Gozde Kutlu

**Affiliations:** 1 Department of Nutrition and Dietetics, Hamidiye Faculty of Health Sciences, University of Health Sciences, 34668 İstanbul, Türkiye; zehrayaman1234@gmail.com (Z.Y.); kubrafeyza.erol@sbu.edu.tr (K.F.E.); 2 Department of Gastronomy and Culinary Arts, Faculty of Fine Arts, Design and Architecture, Ankara Medipol University, 06050 Ankara, Türkiye

**Keywords:** edible flowers, phenolic compounds, amino acid profile, enzyme inhibition, INFOGEST simulated digestion, cytotoxic activity

## Abstract

Edible flowers are increasingly recognized as sources of bioactive compounds; however, comparative evidence on their nutritional composition, gastrointestinal bioaccessibility, and biological activities remains limited. This study aimed to comprehensively evaluate the proximate and mineral composition, total and individual phenolic profiles, antioxidant capacity, and amino acid composition of *Jasminum officinale*, *Althaea officinalis*, and *Viola odorata* flowers before and after simulated gastrointestinal digestion, with particular emphasis on the bioaccessibility of phenolic compounds and amino acids. In addition, the enzyme inhibitory activities and in vitro effects of the flower extracts on the viability of HEK-293, Caco-2, and MiaPaCa-2 cells were investigated. The proximate composition varied among the edible flowers, with dry matter, crude protein, crude fat, ash, and carbohydrate contents ranging from 8.35 to 11.43%, 3.80 to 4.74%, 2.55 to 4.35%, 9.57 to 11.88%, and 69.40 to 79.94%, respectively. The mineral composition also varied considerably among the edible flowers, with calcium (936.32–1463.23 mg/100 g dw) and potassium (1637.74–1857.46 mg/100 g dw) being the predominant minerals. *J. officinale* was particularly rich in magnesium (342.45 mg/100 g dw), *A. officinalis* in calcium and zinc (1463.23 and 4.62 mg/100 g dw, respectively), and *V. odorata* in phosphorus, iron, and manganese (253.56, 18.39, and 5.78 mg/100 g dw, respectively). Before digestion, *J. officinale* exhibited the highest total phenolic content (884.00 mg GAE/100 g) and antioxidant capacity, with a phenolic bioaccessibility index of 60.89%. Simulated digestion generally reduced the phenolic content and antioxidant activity. *V. odorata* was particularly rich in protocatechuic acid, catechin, chlorogenic acid, ferulic acid, and ellagic acid. *A. officinalis* showed the strongest in vitro inhibitory activity against α-glucosidase, α-amylase, acetylcholinesterase, and lipoxygenase, whereas *J. officinale* exhibited the strongest butyrylcholinesterase inhibition. Extracts displayed differential cytotoxic effects against Caco-2 and MiaPaCa-2 cells, while effects on HEK-293 cells were comparatively weaker. Overall, these findings demonstrate species-specific nutritional and bioactive profiles and support further investigation of edible flowers as multifunctional food ingredients.

## 1. Introduction

Phytochemicals have attracted increasing scientific interest because of their diverse biological activities and their potential to promote human health while reducing the risk of chronic diseases. These naturally occurring compounds, including phenolic acids, flavonoids, alkaloids, tannins, saponins, terpenoids, and other secondary metabolites, are widely distributed in edible plants and have been associated with numerous physiological benefits [1]. In parallel with the growing consumer demand for functional foods, plant-derived bioactive compounds are increasingly being explored as sustainable alternatives to synthetic food additives and therapeutic agents owing to their multifunctional biological properties [1,2,3,4].

Among these biological activities, the potential of phytochemicals to modulate metabolic disorders has received particular attention. Diabetes mellitus, one of the most prevalent chronic metabolic diseases worldwide, is characterized by persistent hyperglycemia resulting from impaired insulin secretion, insulin action, or both. Numerous plant-derived phytochemicals, especially polyphenols and flavonoids, have demonstrated antidiabetic potential through multiple mechanisms, including enhancement of insulin sensitivity, attenuation of intestinal glucose absorption, and inhibition of carbohydrate-hydrolyzing enzymes such as α-amylase and α-glucosidase [5]. Consequently, natural enzyme inhibitors have emerged as promising dietary strategies for controlling postprandial hyperglycemia while minimizing the adverse effects commonly associated with synthetic drugs [6,7].

Beyond glucose metabolism, increasing evidence indicates that plant phytochemicals exert neuroprotective effects through multiple molecular pathways. Natural compounds such as flavonoids, phenolic acids, alkaloids, and glycosides have been reported to alleviate neurodegenerative processes by inhibiting acetylcholinesterase (AChE) and butyrylcholinesterase (BChE), thereby preserving cholinergic neurotransmission and cognitive function [6,7,8]. Likewise, their anti-inflammatory properties have attracted considerable attention because chronic inflammation and oxidative stress are closely associated with the development and progression of metabolic and neurodegenerative disorders. Excessive production of reactive oxygen species (ROS) stimulates inflammatory signaling pathways and promotes the formation of pro-inflammatory mediators. Therefore, inhibition of lipoxygenase (LOX), a key enzyme involved in the biosynthesis of inflammatory lipid mediators, has become an important indicator for evaluating the anti-inflammatory potential of natural bioactive compounds [9].

Edible flowers have traditionally been consumed not only as culinary ingredients but also as medicinal plants in many cultures. More recently, they have attracted growing interest as functional food ingredients because they simultaneously improve the sensory characteristics of foods while providing valuable nutrients and bioactive phytochemicals [10]. Edible flowers are recognized as important sources of vitamins, minerals, amino acids, and phenolic compounds, which contribute substantially to their antioxidant capacity and other health-promoting properties [11]. Emerging evidence suggests that their regular consumption may contribute to the prevention of chronic diseases, including cardiovascular diseases and certain cancers, largely because of their rich phytochemical composition and antioxidant potential [12].

Among edible flowers, *Jasminum officinale* L. (Oleaceae) has attracted considerable interest owing to its abundance of phenolic compounds, flavonoids, alkaloids, tannins, saponins, and terpenoids, which have been associated with antioxidant, anti-inflammatory, antimicrobial, antidiabetic, and anticancer activities [12,13]. Likewise, *Althaea officinalis* L. (marshmallow; Malvaceae) has been extensively used in traditional medicine for the treatment of respiratory and gastrointestinal disorders as well as skin injuries. Contemporary pharmacological studies have demonstrated that its biological activities, including antioxidant, antibacterial, anti-inflammatory, antiulcer, immunomodulatory, wound-healing, and antitussive effects, are largely attributed to its diverse phytochemical composition [14,15]. Similarly, *Viola odorata* L. (Violaceae), commonly known as sweet violet, has long been utilized in traditional medicine throughout Asia and the Middle East. Its flowers contain abundant flavonoids, phenolic compounds, alkaloids, triterpenoids, and cyclotides, which have been associated with antioxidant, anti-inflammatory, antibacterial, antidiabetic, diuretic, and anticancer activities, supporting its therapeutic use in respiratory diseases, diabetes, headaches, and inflammatory disorders [16,17,18].

Although several edible flower species have been reported to contain diverse phenolic compounds with considerable antioxidant potential, existing studies have predominantly focused on their phytochemical composition under undigested conditions [1,11,16,18,19,20,21,22,23,24,25]. However, the biological relevance of these phytochemicals depends not only on their abundance in the plant matrix but also on their stability and bioaccessibility during gastrointestinal digestion. Although a limited number of studies have evaluated the effects of INFOGEST in vitro digestion on total phenolic content and antioxidant activity in edible flowers, digestion-induced changes in individual phenolic profiles have not been reported for *J. officinale*, *A. officinalis*, or *V. odorata* and remain largely unexplored across edible flowers. Similarly, despite the fundamental role of gastrointestinal protein hydrolysis in determining amino acid bioaccessibility, digestion-induced changes in amino acid composition have not been investigated in these species and have only rarely been examined in edible flowers overall.

Based on these knowledge gaps, we hypothesized that edible flower species possessing distinct phytochemical compositions would exhibit species-specific changes in phenolic and amino acid profiles during standardized INFOGEST gastrointestinal digestion, leading to differences in bioaccessibility and biological activities. Therefore, this study comparatively evaluated *J. officinale*, *A. officinalis*, and *V. odorata* by determining changes in total phenolic content, HPLC-DAD phenolic profiles, antioxidant capacity, and amino acid composition before and after in vitro digestion. In addition, in vitro enzyme inhibitory activities (α-amylase, α-glucosidase, AChE, BChE, and LOX) together with cytotoxic activity were investigated to provide a comprehensive assessment of their functional potential.

## 2. Materials and Methods

### 2.1. Plant Materials

Fresh flowers of jasmine (*Jasminum officinale* L.) were obtained from potted plants in Muratpaşa, Antalya, Türkiye, in July 2023, whereas fresh flowers of marshmallow (*Althaea officinalis* L.) and sweet violet (*Viola odorata* L.) were collected from field-grown plants in Kahramankazan, Ankara, Türkiye, in July and April 2023, respectively, during their respective flowering seasons. The flowers were manually sorted to remove damaged tissues and foreign materials, washed thoroughly with tap water followed by distilled water, and gently blotted dry with paper towels. The samples were then shade-dried at room temperature (approximately 20–25 °C) in the dark in a well-ventilated environment for 5 days until a constant weight was reached. Subsequently, the dried flowers were ground into a fine powder using a laboratory mill and passed through a 40-mesh sieve to obtain a uniform particle size. The powdered samples were transferred into polyethylene bags, protected from light, and stored at −18 °C until extraction and further analyses [10]. Moreover, the botanical identification of the edible flower species was conducted by Prof. Hüseyin Fakir (Faculty of Forestry, Isparta University of Applied Sciences, Türkiye) based on the morphological characteristics of the plant specimens.

### 2.2. Ultrasound-Assisted Extraction

Ultrasound-assisted extraction (UAE) was performed according to the method of Erol et al. [3] with minor modifications. Briefly, 1 g of powdered flower sample was mixed with 20 mL of ethanol/water (80:20, *v*/*v*) (solid-to-solvent ratio, 1:20, *w*/*v*) and homogenized using an Ultra-Turrax homogenizer (Daihan HG-15D, Gang-Won-Do, South Korea) at 10,000 rpm for 3 min. The homogenized mixture was subsequently extracted in an ultrasonic bath (WiseClean DH.WUC.D10H, Wertheim, Germany) at 100 W and 30 °C for 6 min. After extraction, the suspensions were centrifuged at 5000× *g* for 10 min, and the supernatants were filtered through a 0.45 μm membrane filter. The concentrated extracts were then frozen at −18 °C for 12 h and subsequently freeze-dried using a lyophilizer (Christ β 1,8-LSC plus, Martin Christ GmbH, Osterode am Harz, Germany). The resulting dried extracts were gently ground to a fine powder using a porcelain mortar (ISOLAB Laborgeräte GmbH, Wertheim, Germany) and stored in tightly closed containers at 4 °C in the dark until further analysis. For each flower species, three independent samples (1 g each) were separately subjected to the complete extraction procedure, resulting in three independent extracts. Subsequent analyses were performed on these extracts as analytical replicates. The extraction yields obtained by UAE were 11.43 ± 0.21%, 8.89 ± 0.15%, and 10.53 ± 0.15% for *J. officinale*, *A. officinalis*, and *V. odorata*, respectively.

### 2.3. Proximate Composition and Mineral Content

Moisture, crude protein, crude fat, and ash contents of the edible flowers were determined according to Kutlu and Erol [10]. Total carbohydrate content was calculated by difference:

Carbohydrate (%) = 100 − [Moisture (%) + Ash (%) + Protein (%) + Fat (%)](1)

Mineral contents (Ca, K, Mg, P, Na, Fe, Zn, Cu, and Mn) were determined according to Erol et al. [2], with slight modifications. Briefly, approximately 0.25 g of sample was digested with concentrated HNO_3_ (69%) and H_2_O_2_ (30%) using a microwave digestion system (Berghof, Eningen, Germany). Samples were pre-digested at room temperature for 10 min before microwave digestion. Following digestion, the solutions were diluted with deionized water and analyzed using an atomic absorption spectrophotometer (AAnalyst 800, PerkinElmer, Waltham, MA, USA). Quantification was performed using element-specific hollow cathode lamps (PerkinElmer, Waltham, MA, USA) and external calibration curves prepared from certified standards. For each flower species, three independent samples were separately subjected to the complete digestion and mineral determination procedure (*n* = 3). Each sample was analyzed in duplicate to assess analytical repeatability, and results were expressed as mg/100 g dry weight.

### 2.4. Total Phenolic Content (TPC) and Antioxidant Activity

TPC was determined using the Folin–Ciocalteu method according to Aparı et al. [26], with slight modifications. Briefly, 20 μL of each edible flower extract was dispensed into a 96-well microplate, followed by the addition of 100 μL of 10% (*v*/*v*) Folin–Ciocalteu reagent. After allowing the reaction to proceed for 1 min, 80 μL of 7.5% (*w*/*v*) sodium carbonate solution was added, and the reaction mixture was incubated in the dark at room temperature (25 °C) for 30 min. Subsequently, the absorbance was recorded at 765 nm using a microplate spectrophotometer (Bio-Tek, ELX808IU, Winooski, VT, USA). Gallic acid (Sigma-Aldrich, St. Louis, MO, USA) was used as the reference standard, and the results were expressed as milligrams of gallic acid equivalents per 100 g of dry extract (mg GAE/100 g DW). The calibration curve was prepared using gallic acid concentrations ranging from 0.01 to 0.10 mg/mL (0.01, 0.02, 0.03, 0.04, 0.06, 0.07, 0.08, and 0.10 mg/mL), and the calibration equation was y = 9.7692x + 0.0115, with a coefficient of determination (R^2^) of 0.996.

The antioxidant activity of the lyophilized edible flower extracts was evaluated using three complementary in vitro assays, namely 2,2′-Azinobis(3-ethylbenzothiazoline-6-sulfonic acid) (ABTS) radical cation scavenging, CUPRAC (cupric ion reducing antioxidant capacity), and DPPH radical scavenging assays. Since each assay is based on different reaction mechanisms, the combined use of these methods provides a more comprehensive assessment of the antioxidant potential of plant extracts. The antioxidant capacities obtained from all assays were expressed as µmol Trolox equivalents per gram of dry matter (µmol TE/g DM).

The CUPRAC activity of the lyophilized extracts was determined according to the applied method of Kutlu & Erol et al. [10] with slight modifications. Briefly, 10 mM of CuCl_2_ solution, 7.5 mM of neocuproine solution, 1 M of ammonium acetate buffer (pH 7.0), and the edible flower extract (0.5 mL) were mixed and vortexed thoroughly. After incubation in the dark for 30 min at room temperature, the absorbance was measured at 450 nm using a UV–Vis spectrophotometer (Shimadzu, UV-1800, Kyoto, Japan). Antioxidant capacity was calculated from a Trolox calibration curve prepared using Trolox concentrations ranging from 0.025 to 0.8 mg/mL (0.025, 0.05, 0.1, 0.2, 0.3, 0.4, 0.5, 0.6, 0.7, and 0.8 mg/mL). The calibration equation was y = 1.4696x − 0.0345, with a R^2^ of 0.9977.

The DPPH radical scavenging activity of the lyophilized extracts was determined according to Yasar et al. [11] with slight modifications. An aliquot of the edible flower extract (0.1 mL) was mixed with DPPH solution (4.9 mL, 0.1 mM), vortexed, and incubated in the dark for 30 min at room temperature. The decrease in absorbance was measured at 517 nm using a UV–Vis spectrophotometer (Shimadzu, UV-1800, Kyoto, Japan). The antioxidant activity was calculated using a Trolox standard curve prepared using Trolox concentrations ranging from 0.025 to 0.8 mg/mL (0.025, 0.05, 0.1, 0.2, 0.3, 0.4, 0.5, 0.6, 0.7, and 0.8 mg/mL). The calibration equation was y = 1.631x − 0.0024, with an R^2^ of 0.9992.

The ABTS•^+^ radical cation was generated by reacting 7 mM ABTS solution with 2.45 mM potassium persulfate and allowing the mixture to stand in the dark at room temperature for 12–16 h before use. The resulting stock solution was diluted with ethanol to obtain an absorbance of approximately 0.70–0.90 at 734 nm. The edible flower extract (100 μL) was then mixed with the working ABTS solution, vortexed, and incubated in the dark at room temperature for 5–6 min before measuring the absorbance at 734 nm and calculating the antioxidant capacity using a Trolox calibration curve prepared at concentrations of 0.01, 0.025, 0.05, 0.075, 0.1, and 0.2 mg/mL. The calibration equation was y = 3.4444x + 0.054, with an R^2^ coefficient of 0.992.

### 2.5. Phenolic Acid Profile by HPLC–DAD

Phenolic compounds were quantified in undigested extracts and IN fractions obtained following INFOGEST digestion. Analysis was performed according to Yasar et al. [11], with minor modifications, using an HPLC–DAD system (Shimadzu, Kyoto, Japan) equipped with an Inertsil ODS C18 column (250 × 4.6 mm, 5 μm) maintained at 40 °C. The mobile phase consisted of 0.1% acetic acid in water (A) and 0.1% acetic acid in acetonitrile (B), delivered at 1.0 mL/min under gradient conditions: 10% B (0–2 min), 30% B (2–27 min), 90% B (27–50 min), and 100% B (50–60 min), followed by re-equilibration to the initial conditions until 63 min. Compounds were identified by comparison with authentic standards and quantified using individual external calibration curves. For the undigested samples and IN phase, phenolic acid concentrations were expressed as µg/g of UAE lyophilized extract.

### 2.6. INFOGEST Simulated Gastrointestinal Digestion and Bioaccessibility

Simulated gastrointestinal digestion was performed according to the standardized INFOGEST static protocol [27], with minor modifications. The simulated oral, gastric, and intestinal fluids were prepared using the stock solutions described by Minekus et al. (2014) [27]. Simulated salivary fluid (SSF), simulated gastric fluid (SGF), and simulated intestinal fluid (SIF) were prepared from the stock solutions of KCl (0.5 mol/L), KH_2_PO_4_ (0.5 mol/L), NaHCO_3_ (1.0 mol/L), NaCl (2.0 mol/L), MgCl_2_·6H_2_O (0.15 mol/L), and (NH_4_)_2_CO_3_ (0.5 mol/L). For the oral phase, 15.1, 3.7, 6.8, 0.5, 0.06, and 0.09 mL of KCl, KH_2_PO_4_, NaHCO_3_, MgCl_2_·6H_2_O, (NH_4_)_2_CO_3_, and HCl stock solutions, respectively, were used. For the gastric phase, 6.9, 0.9, 12.5, 11.8, 0.4, 0.5, and 1.3 mL of KCl, KH_2_PO_4_, NaHCO_3_, NaCl, MgCl_2_·6H_2_O, (NH_4_)_2_CO_3_, and HCl stock solutions, respectively, were used. For the intestinal phase, 6.8, 0.8, 42.5, 9.6, 1.1, and 0.7 mL of KCl, KH_2_PO_4_, NaHCO_3_, NaCl, MgCl_2_·6H_2_O, and HCl stock solutions, respectively, were used. The required volumes of each stock solution were used to prepare SSF, SGF, and SIF, and each digestion fluid was brought to a final volume of 400 mL with distilled water. The final pH values were adjusted to 7.0, 3.0, and 7.0 for the oral, gastric, and intestinal phases, respectively. All simulated digestive fluids and enzyme solutions were preheated to 37 °C before use. The activities of α-amylase, pepsin, and pancreatin used in the in vitro gastrointestinal digestion experiments were verified prior to initiating the digestion experiments. Rather than relying solely on the nominal activity values reported by the manufacturers, the enzymatic activities were experimentally checked before use. The enzyme preparations used in the experiments were either newly opened or freshly prepared, thereby minimizing the potential effects of activity losses associated with prolonged storage on the experimental results. The activities determined experimentally were compared with the target enzyme activities recommended in the INFOGEST protocol, and the amounts of enzymes used in the digestion experiments were calculated based on the verified activity values.

For the oral phase, 0.5 g of each dried and finely milled edible flower sample was mixed with 3.5 mL of SSF, 0.5 mL of α-amylase solution (75 U/mL), 25 µL of 0.3 M CaCl_2_ solution, and distilled water to a final volume of 5 mL. The mixture was incubated at 37 °C for 2 min in a shaking water bath at 100 rpm. Following the oral phase, the samples were centrifuged at 4500 rpm for 10 min at 4 °C, and the supernatants were collected and stored at −20 °C.

Following the oral phase, 6 mL of SGF, 1.28 mL of pepsin solution (2000 U/mL), and 4 µL of 0.3 M CaCl_2_ solution were added. The pH was adjusted to 3.0 using 1 M HCl, and the final volume was adjusted to 12.4 mL with distilled water. The mixture was incubated at 37 °C for 2 h in a shaking water bath at 100 rpm. Following gastric digestion, the samples were centrifuged at 4500 rpm for 10 min at 4 °C, and the supernatants were collected and stored at −20 °C.

For the intestinal phase, 7.7 mL of SIF, 3.5 mL of pancreatin solution (100 U/mL), 1.75 mL of bile salt solution (prepared at a concentration of 160 mM), and 28 µL of 0.3 M CaCl_2_ solution were added to the gastric digest. The bile salt solution was prepared by dissolving 250 mg of bile salts in 10 mL of SIF. The concentration of 160 mM refers to the concentration of the bile salt solution prepared for use in the intestinal phase and does not represent the final bile salt concentration in the intestinal digestion mixture. The pH was adjusted to 7.0 using 1 M NaOH, and the final volume was adjusted to 26 mL with distilled water. The mixture was incubated at 37 °C for 2 h in a shaking water bath. During the intestinal phase, a dialysis tube was completely immersed in the liquid phase, and the beaker was tightly sealed with Parafilm. Dialysis tubing with a molecular weight cut-off of 12,000 Da was used to separate the dialyzable and non-dialyzable fractions. Before use, the dialysis tubing was washed with 0.09% NaCl solution on both the inner and outer surfaces. The tubing was then filled with the required amount of 0.1 M NaHCO_3_ solution, and both ends were tightly sealed before being immersed in the intestinal digestion mixture. At the end of the simulated intestinal digestion, the fraction inside the dialysis tube was collected as the IN (dialyzable) fraction, whereas the fraction outside the dialysis tube was collected as the OUT (non-dialyzable) fraction. At the end of the intestinal digestion stage, Pefabloc SC (4-(2-aminoethyl) benzenesulfonyl fluoride hydrochloride) was used to terminate the activity of proteolytic enzymes. Pefabloc SC was prepared as a 0.1 M stock solution and added to the intestinal digesta at a ratio of 50 μL per 1 mL of intestinal digesta, resulting in a final concentration of 5 mM. Following the addition of Pefabloc SC, the samples were thoroughly mixed to ensure homogeneity, and the enzymatic reactions were terminated. This procedure was performed in accordance with the procedure recommended in the INFOGEST 2.0 protocol for terminating intestinal digestion. Importantly, no centrifugation, filtration, dialysis, or other fractionation step was performed between the successive digestion phases. At the end of each digestion phase, the entire digestion mixture (i.e., the unfractionated digesta) was directly carried forward to the subsequent phase.

A digestion blank without edible flower sample was prepared and processed in parallel with the samples under the same oral, gastric, and intestinal digestion conditions to determine potential analytical contributions arising from the simulated digestive fluids and digestion reagents. The blank fractions obtained from the corresponding digestion stages were used for background correction in the respective analyses. The corresponding digestion blank values were subtracted from the analytical results of the digested samples to correct for the contribution of the digestion reagents and simulated digestive fluids. Following each digestion stage, the collected fractions were frozen at −80 °C and subsequently freeze-dried (Martin Christ, Beta 1–8 LSC plus, Osterode am Harz, Germany) prior to analysis. The undigested samples were used as baseline controls and were compared with the results obtained after the intestinal digestion phase. The dilution factors applied during the analyses were also taken into account in the calculation of the final results.

TPC, antioxidant activity, phenolic composition, and amino acid profile were determined in the undigested and IN fractions. The relative bioaccessibility index (BI) was calculated as the fraction relative to its concentration in the corresponding UAE extract (undigested sample) and expressed as a percentage. This index was used as a comparative measure of compound recovery following simulated gastrointestinal digestion rather than as a direct mass-balance-based estimate of bioaccessibility.

### 2.7. Amino Acid Composition

Amino acid profiles of the undigested samples and IN fractions were determined by HPLC, according to the established procedure with some modifications [28]. The amino acid composition of the samples was determined by high-performance liquid chromatography (HPLC) following pre-column derivatization with phenylisothiocyanate (PITC). Unless otherwise stated, all solutions were freshly prepared on the day of analysis. A 0.1 N hydrochloric acid (HCl) solution was prepared by adding 8.28 mL of HCl to a 1 L volumetric flask and making up to volume with deionized water. A 6 N HCl solution was prepared by adding 496.8 mL of HCl to a 1 L volumetric flask and making up the volume with deionized water. The derivatization reagent was prepared by mixing 1.2 mL of PITC with 100 mL of acetonitrile. A 0.02 M ammonium acetate solution was prepared by dissolving 0.31 g of ammonium acetate in deionized water and adjusting the final volume to 200 mL. The acetonitrile: methanol (ACN:MeOH) mixture was prepared by mixing 100 mL of acetonitrile, 50 mL of methanol, and 20 mL of triethylamine without further volume adjustment.

Amino acid stock standard solutions were prepared at a concentration of 100 µg/mL by accurately weighing 10 mg of each amino acid into separate 100 mL volumetric flasks, dissolving the standards in 0.1 N HCl, and making up to volume with the same solvent. Working standard solutions were prepared by appropriate dilution of the stock solutions.

For sample preparation, 0.1–1.0 g of thoroughly homogenized sample was weighed into a 50 mL screw-capped digestion bottle, and 20 mL of 6 N HCl was added. The bottle was flushed with nitrogen, tightly sealed, and hydrolyzed at 110 °C for 24 h. Following hydrolysis, the samples were cooled to room temperature and filtered through conventional filter paper. An aliquot of 0.2 mL of the filtrate was transferred to a glass tube and evaporated to dryness under a nitrogen stream at 50 °C. Subsequently, 0.5 mL of acetonitrile was added, and the evaporation step was repeated. Approximately 0.5 mL of the ACN: MeOH mixture and 0.1 mL of the PITC derivatization reagent were then added to the residue, and derivatization was performed at 40 °C for 30 min. After derivatization, the solvent was evaporated under a nitrogen stream at 40 °C. The residue was redissolved in 0.2 mL of acetonitrile and evaporated again under nitrogen. Finally, 5 mL of 0.02 M ammonium acetate solution was added, and the resulting solution was filtered through a 0.2 µm membrane filter prior to injection into the UFLC system (Shimadzu Corporation, Kyoto, Japan). The amount of sample used for analysis was adjusted according to the expected concentration of the target amino acids in the sample matrix.

Chromatographic separation was performed on an Agilent Eclipse XDB-C18 column (5 µm, 4 × 150 mm) (Agilent, Santa Clara, CA, USA) using a reversed-phase gradient elution system. Mobile phase A consisted of a phosphate buffer prepared by dissolving 0.78 g of sodium dihydrogen phosphate dihydrate and 0.88 g of disodium hydrogen phosphate dihydrate in deionized water and making the final volume up to 1 L. The pH of the buffer was adjusted to 6.8–6.9, followed by filtration prior to use. Acetonitrile was used as mobile phase B. The column temperature was maintained at 40 °C, and detection was performed at 254 nm using a UV detector. The injection volume and flow rate were 10 µL and 1.0 mL/min, respectively. The gradient program was initiated with 100% mobile phase A and 0% mobile phase B, followed by a gradual increase in the proportion of mobile phase B to 15% at 13.0 min, 25% at 22.0 min, 30% at 26.0 min, and 60% at 28.0 min. The initial conditions were restored at 38.01 min. Amino acids were identified by comparing their retention times with those of the corresponding analytical standards and quantified using external calibration curves. Calibration curves were considered acceptable when the coefficient of determination (R^2^) was ≥0.99. The concentrations of amino acids in the samples were calculated from the respective calibration equations, taking into consideration the sample weight, dilution factors, and final solution volumes. The difference between parallel determinations was required to be ≤15%.

Tryptophan was determined separately by HPLC using a reversed-phase Lichospher 60 RP-select B column (5 µm; LiChroCART 250-4 HPLC cartridge, Merck KGaA, Darmstadt, Germany). A 0.1 N HCl solution was prepared by adding 8.28 mL of HCl to a 1 L volumetric flask, making up to volume with deionized water, and adjusting the pH to 6.3. A 5 N NaOH solution was prepared by dissolving 200 g of sodium hydroxide in deionized water and making the final volume up to 1 L. A tryptophan stock standard solution (100 µg/mL) was prepared by accurately weighing 10 mg of tryptophan into a 100 mL volumetric flask, dissolving it in an appropriate volume of the pH 6.3 HCl solution, and making up to volume with the same solvent. Working standards were prepared by appropriate dilution of the stock solution.

For tryptophan analysis, 0.1–1.0 g of thoroughly homogenized sample was weighed into a 50 mL screw-capped bottle, and 20 mL of 5 N NaOH was added. The bottle was flushed with nitrogen, tightly sealed, and hydrolyzed at 120 °C for 12 h. After hydrolysis, the samples were cooled to room temperature and filtered through conventional filter paper. An aliquot of 1 mL of the filtrate was transferred to a suitable vessel, and 60 mL of deionized water was added. The pH was adjusted to 6.3 using 0.1 N HCl, and the solution was made up to a final volume of 100 mL with deionized water. The solution was subsequently filtered through a 0.45 µm membrane filter and injected into the HPLC system.

The mobile phase was prepared by dissolving 3 g of ammonium acetate in approximately 900 mL of deionized water. The pH was adjusted to 3.0, followed by the addition of 100 mL of acetonitrile, and the mobile phase was thoroughly mixed and filtered before use. Tryptophan was detected using a fluorescence detector with excitation and emission wavelengths of 280 and 340 nm, respectively. The injection volume was 10 µL, the flow rate was 1.0 mL/min, and the total analysis time was 10 min. Tryptophan was identified based on its retention time relative to the corresponding standard and quantified using an external calibration curve. Linearity was accepted at R^2^ ≥ 0.99, and the tryptophan concentration was calculated from the calibration equation by considering the sample weight, dilution factors, and final solution volume. The difference between parallel determinations was required to be ≤15%.

### 2.8. In Vitro Enzyme Inhibitory Activity of the Edible Flowers

The inhibitory activities of lyophilized edible flower extracts against α-glucosidase and α-amylase were determined according to Erol et al. [3], respectively, with minor modifications. Briefly, 50 μL of extract at different concentrations prepared in distilled water was pre-incubated with 25 μL of α-amylase (0.5 U/mL) for 15 min at 37 °C. The enzymatic reaction was initiated by adding 50 μL of 0.5% soluble starch prepared in 20 mM phosphate buffer (pH 6.9). After incubation for 10 min at 37 °C, the reaction was terminated by adding 600 μL of 3,5-dinitrosalicylic acid (DNS) reagent, followed by heating at 100 °C for 10 min. After cooling to room temperature, absorbance was measured at 540 nm using a microplate reader (Bio-Tek, ELX808IU, Winooski, VT, USA). Acarbose was used as the positive control. Extract concentrations of 0.125, 0.25, 0.50, 0.75, 1.0, 1.5, 2.0, and 4.0 mg/mL were tested. IC_50_ values were calculated by nonlinear regression analysis of the concentration–response curves.

Briefly, 25 μL of extract at different concentrations was mixed with 25 μL of α-glucosidase (0.5 U/mL) and pre-incubated at 25 °C for 10 min. The reaction was initiated by adding 25 μL of 0.5 mM p-nitrophenyl-α-D-glucopyranoside (pNPG), followed by incubation at 37 °C for 30 min. The reaction was terminated by adding 100 μL of 0.2 M sodium carbonate, and absorbance was measured at 405 nm using a microplate reader (Bio-Tek, ELX808IU, USA). Acarbose was used as the positive control. Extract concentrations of 0.125, 0.25, 0.50, 0.75, 1.0, 1.5, 2.0, and 4.0 mg/mL were tested. IC_50_ values were calculated by nonlinear regression analysis of the concentration–response curves.

Sample blanks containing the respective extract but without enzyme were included, where applicable, to correct for background absorbance arising from the intrinsic color of the extracts. Enzyme and substrate controls were also included to determine the baseline enzymatic activity. All measurements were performed in triplicate.

AChE and BChE inhibitory activities were determined according to Erol et al. [3] using the Ellman method with minor modifications. Acetylcholinesterase obtained from electric eel and butyrylcholinesterase derived from horse serum were used as enzyme sources. Briefly, 20 μL of each edible flower extract (prepared by dilution of a 50 mg/mL stock solution in DMSO) was combined with 140 μL of 0.1 M phosphate buffer (pH 6.8) and 20 μL of either AChE or BChE enzyme solution (5 × 10^−3^ M). Following a pre-incubation period of approximately 10 min, the enzymatic reaction was initiated by adding 10 μL of 3 mM 5,5′-dithiobis-(2-nitrobenzoic acid) (DTNB) together with 10 μL of the appropriate substrate, namely 0.71 mM acetylthiocholine iodide for the AChE assay or 0.2 mM butyrylthiocholine iodide for the BChE assay. After completion of the reaction, absorbance was recorded at 405 nm using a microplate reader (Bio-Tek, ELX808IU, Winooski, VT, USA). Galanthamine hydrobromide was employed as the positive control. The inhibitory activities of the extracts were expressed as IC_50_ values (μg/mL), representing the concentration required to inhibit 50% of enzyme activity.

LOX enzyme inhibition was evaluated according to Alaba and Chichioco-Hernandez [29], with minor modifications. Quercetin solution (3.74 mM, approximately 1.13 mg/mL) was used as the positive control. A quercetin stock solution was prepared at 3.74 mM and serially diluted to obtain the concentrations required for the dose–response analysis. Lipoxygenase solution (8460 U/mL) was prepared in 0.1 M phosphate buffer (pH 8.0), corresponding to a final enzyme activity of 141 U/mL in the 3.0 mL reaction mixture, while linoleic acid was prepared at a concentration of 1.98 mM. The reaction mixture consisted of 50 μL of lipoxygenase solution (8460 U/mL), 2740 μL of phosphate buffer (0.1 M, pH 8.0), and 10 μL of edible flower extract at different concentrations. Following a 5 min pre-incubation at 25 °C, the enzymatic reaction was initiated by adding 200 μL of linoleic acid substrate (1.98 mM). The total reaction volume was 3.0 mL, corresponding to final concentrations of 141 U/mL lipoxygenase and 132 μM linoleic acid in the reaction mixture. The increase in absorbance was monitored at 234 nm every 30 s for 5 min using a UV–Vis spectrophotometer (Shimadzu UV-1800, Kyoto, Japan). Control reactions were prepared by replacing the extract with phosphate buffer under identical experimental conditions. The percentage inhibition of lipoxygenase activity was calculated by comparing the absorbance of the sample with that of the corresponding control. Dose–response curves were constructed using the tested extract concentrations, and the IC_50_ values, defined as the concentration required to inhibit 50% of lipoxygenase activity, were determined by linear regression analysis.

For all enzyme inhibition assays, sample blanks were prepared to account for potential absorbance interference arising from the intrinsic color of the extracts. Enzyme and substrate controls were also included to monitor enzyme activity and to identify any nonspecific absorbance changes caused by interactions of the extracts with components other than the enzyme or substrate. Positive controls included acarbose for α-amylase and α-glucosidase, galanthamine hydrobromide for AChE and BChE, and quercetin for LOX. All sample, control, and blank groups were evaluated under the same experimental conditions. Inhibition percentages were calculated relative to the corresponding control using blank-corrected absorbance values. Dose–response curves were generated from the concentration–response data obtained at different extract concentrations, and IC_50_ values were determined by nonlinear regression analysis. Measurements were performed in triplicate.

### 2.9. MTT-Based Cell Metabolic Viability 

The cytotoxic effects of the lyophilized edible flower extracts were evaluated using the 3-(4,5-dimethylthiazol-2-yl)-2,5-diphenyltetrazolium bromide (MTT) assay with minor modificatiıns [6]. Two human cancer cell lines, Caco-2 (human colorectal adenocarcinoma, ATCC HTB-37) and MiaPaCa-2 (human pancreatic carcinoma, ATCC CRL-1420), together with adenovirus-transformed HEK-293 (human embryonic kidney, ATCC CRL-1573) cell line, were obtained from the American Type Culture Collection (ATCC, Manassas, VA, USA) and employed to assess the in vitro cytotoxic potential of the extracts. All cell lines were maintained in fresh DMEM culture medium supplemented with 10% fetal bovine serum (Sigma-Aldrich, St. Louis, MO, USA), 2 mM L-glutamine, and 100 U/mL penicillin/streptomycin under standard cell culture conditions at 37 °C in a humidified atmosphere containing 5% CO_2_ and 95% air. The culture medium was refreshed every 3 days during the incubation period. Once the cells reached 60–80% confluence, they were detached by trypsinization, and cell counting was performed using trypan blue staining and an automated cell counter (Bio-Rad, Hercules, CA, USA).

For the cytotoxicity assay, cells were seeded into 96-well microplates at a density of 1.5 × 10^4^ cells per well and allowed to attach for 24 h. Subsequently, the culture medium was replaced with fresh medium containing the edible flower extracts at final concentrations of 1, 50, 100, 250, 500, and 1000 μg/mL. The lyophilized edible flower extracts were dissolved in DMSO to prepare stock solutions and appropriately diluted with the corresponding culture medium to obtain the final concentrations. Untreated cells maintained in the corresponding culture medium served as the negative control, whereas cells treated with the same final concentration of DMSO present in the extract-treated wells, without the extract, served as the vehicle control. Each treatment condition was tested in triplicate wells, and the experiment was performed in two independent experiments.

Following 24 h of treatment, cell viability was determined using the MTT colorimetric assay. Briefly, MTT solution (5 mg/mL in PBS, prepared and filtered before use) was added to each well under light-protected conditions and incubated at 37 °C for 3 h to allow metabolically active cells to reduce MTT to insoluble formazan crystals. The culture medium was subsequently removed, and 100 μL of dimethyl sulfoxide (DMSO) was added to each well to dissolve the formed formazan crystals. The plates were incubated for 10 min at 37 °C in a CO_2_ incubator. Absorbance was then measured at 590 nm using a microplate reader (Agilent BioTek Epoch, Santa Clara, CA, USA).

Cell-free wells containing the MTT solution, but no cells, were used as the blank control for background correction. The absorbance values obtained from the blank wells were subtracted from the corresponding sample and control absorbance values. The extract–MTT interference control values were also considered when evaluating potential absorbance contributions arising from direct interactions between the extracts and the MTT reagent.

The viability of the negative control cells was defined as 100%, and cell viability was calculated according to the following equation:

% Cell viability = [(AD_590_ of sample − AD_590_ of blank)/(AD_590_ of control − AD_590_ of blank)] × 100

In this equation, sample absorbance represents the absorbance measured at 590 nm for cells treated with the respective extract concentration; blank absorbance represents the background absorbance measured in cell-free wells containing the MTT solution; and control absorbance represents the absorbance measured for untreated control cells. Thus, all sample results were blank-corrected and normalized to the untreated negative control, which was considered to represent 100% cell viability. The extract–MTT interference controls were evaluated together with the corresponding sample and control groups to identify potential absorbance contributions from the extracts independent of cellular metabolic activity.

The concentration-dependent effects of the edible flower extracts were evaluated using dose–response curves generated from the tested concentrations (1, 50, 100, 250, 500, and 1000 μg/mL). IC_50_ values, defined as the concentration required to reduce cell viability by 50% relative to the untreated control, were calculated from the concentration–response data. Cell viability was assessed exclusively using the MTT assay; no additional microscopic viability analysis was performed. Each treatment condition was tested in triplicate wells, and the experiment was performed in two independent experiments. The three technical wells within each independent experiment were averaged and were not treated as independent statistical observations.

### 2.10. Statistical Analysis

Statistical analyses were carried out using JMP 5.0.1 (Version 5.0.1a, SAS Institute, Inc., Cary, NC, USA). The number and type of replicates for each analysis are specified in the corresponding methodological sections. Results are expressed as mean ± standard deviation. One-way analysis of variance (ANOVA) followed by Tukey’s multiple comparison test was used to determine significant differences among samples. Differences were considered statistically significant at *p* < 0.05.

## 3. Results and Discussion

### 3.1. Proximate and Mineral Composition of Edible Flowers

The proximate composition and mineral composition of the investigated edible flowers are presented in Table 1. Significant differences (*p* < 0.05) were observed among the three flower species for all proximate composition parameters, highlighting the influence of botanical origin on their nutritional characteristics. Dry matter content was highest in *A. officinalis* (11.43 ± 0.04%), followed by *V. odorata* (10.57 ± 0.09%) and *J. officinale* (8.35 ± 0.14%). Similarly, *A. officinalis* showed the highest crude protein (4.74 ± 0.10%) and ash (11.88 ± 0.04%) contents, whereas *V. odorata* had the highest crude fat content (4.35 ± 0.03%). In contrast, *J. officinale* exhibited the highest calculated carbohydrate content (79.94 ± 0.06%). These interspecific differences indicate distinct nutritional profiles among the edible flowers and may reflect differences in species characteristics and growing conditions. The relatively high protein and ash contents of *A. officinalis*, together with the higher lipid content of *V. odorata*, warrant further consideration in relation to their amino acid and mineral profiles. As carbohydrates were calculated by difference, its variation should be interpreted in relation to the other proximate components.

The mineral composition also differed significantly among the investigated species. Marshmallow flower was particularly rich in calcium (1463.23 mg/100 g dw), potassium (1857.46 mg/100 g dw), and zinc (4.62 mg/100 g dw), whereas sweet violet contained the highest concentrations of phosphorus (253.56 mg/100 g dw), iron (18.39 mg/100 g dw), and manganese (5.78 mg/100 g dw). Copper was detected only in sweet violet (0.42 mg/100 g dw), while it was not detected in jasmine or marshmallow flower. Jasmine, on the other hand, exhibited the highest magnesium (342.45 mg/100 g dw) and sodium (36.35 mg/100 g dw) contents. From a nutritional perspective, all three edible flowers represent valuable sources of essential minerals. Marshmallow flower contained the highest levels of calcium, potassium, and zinc, whereas sweet violet was particularly rich in phosphorus, iron, manganese, and copper. In contrast, jasmine exhibited the highest magnesium and sodium contents. These findings demonstrate that edible flowers can serve as important dietary sources of essential macro- and microelements, thereby contributing to human nutrition beyond their sensory attributes. These findings are consistent with previous reports indicating that edible flowers represent valuable sources of essential minerals and can contribute meaningfully to human nutrition [30]. Beyond their attractive color, aroma, and flavor, the mineral composition of edible flowers is considered one of the most important factors determining their nutritional value and potential application as functional food ingredients. The marked variation in mineral composition among edible flowers is likely associated with species-specific differences in mineral uptake, transport, and accumulation, as well as genetic and physiological characteristics [10,28].

The relatively high concentrations of calcium, potassium, magnesium, iron, zinc, and manganese detected in the edible flowers indicate that these species may contribute to daily micronutrient intake. These minerals are involved in numerous physiological processes, including enzyme activation, maintenance of bone health, oxygen transport, antioxidant defense systems, and cellular metabolism [2,4]. Consequently, the favorable mineral profiles observed in the present study further support the potential use of these edible flowers as functional food ingredients. To the best of our knowledge, no previous studies have comprehensively reported the proximate composition and mineral profiles of *J. officinale*, *A. officinalis*, and *V. odorata* flowers. Consequently, the present findings provide original nutritional data that may serve as a reference for future nutritional, phytochemical, and functional food studies. Overall, the results demonstrate that each edible flower possesses a distinct nutritional profile, with marshmallow flower showing higher protein and selected mineral contents, jasmine exhibiting a higher magnesium content, and sweet violet showing higher iron, phosphorus, and manganese contents. These compositional differences may influence their functional properties and should be considered when selecting edible flowers for specific food and nutraceutical applications.

### 3.2. TPC and Antioxidant Activities Before and After In Vitro Gastrointestinal Digestion

The TPC, antioxidant activities determined by the ABTS, CUPRAC, and DPPH assays, and their corresponding BI following in vitro gastrointestinal digestion are presented in Table 2. Significant differences (*p* < 0.05) were observed among the edible flower species for all antioxidant parameters evaluated before and after digestion. Prior to digestion, jasmine exhibited the highest antioxidant capacity regardless of the analytical method employed, with ABTS, CUPRAC, and DPPH values of 34.21, 27.43, and 42.34 µmol TE/g DM, respectively. Consistently, jasmine also possessed the highest TPC (884.00 mg GAE/100 g), followed by sweet violet (729.40 mg GAE/100 g) and marshmallow flower (663.34 mg GAE/100 g). The highest antioxidant capacity observed for jasmine coincided with its highest TPC; however, this observation does not establish a direct causal relationship between TPC and antioxidant activity. This concurrent pattern is consistent with the potential contribution of phenolic compounds to antioxidant capacity, although the contribution of other antioxidant constituents cannot be excluded [18]. Because the ABTS, DPPH, and CUPRAC assays are based on different reaction mechanisms and may respond differently to individual antioxidant compounds, the antioxidant responses observed in the present study should not be interpreted solely on the basis of TPC.

The relatively high antioxidant responses observed for *J. officinale* are broadly consistent with previous reports. However, direct quantitative comparisons between the present results and those reported in previous studies should be made with caution, because differences in extraction solvents and conditions, extract concentrations, units of expression, sample basis (e.g., dry weight or extract basis), and analytical methods can substantially affect the reported TPC and antioxidant values. Thus, the numerical values reported in the literature are not directly comparable with the present results unless the experimental and reporting conditions are methodologically standardized. Yasar et al. [11] reported TPC values ranging from 44.40 to 50.88 mg GAE/g extract for ethanol–water extracts of jasmine flowers under different extraction conditions. Likewise, Chen et al. [31] demonstrated that jasmine flowers possessed the highest antioxidant capacity among several herbal floral samples, exhibiting ORAC, DPPH, and ABTS values of 4766.1 ± 185.67 μmol TE/g DW, 165.58 ± 6.76 mg QE/g DW, and 573.07 ± 14.90 mg VCE/g DW, respectively. Yasar et al. [11] reported DPPH, CUPRAC, and FRAP values of 20.21, 18.84, and 143.93 mg TE/g, respectively, for *J. officinale* flower extracts. Likewise, Memdueva et al. [15] demonstrated that *A. officinalis* extracts exhibited considerable antioxidant activity, with DPPH values ranging from 72 to 89 μmol TE/L, ABTS radical scavenging activities between 73% and 89%, and FRAP values from 0.83 to 8.09 mg Fe(II)/L. These reports support the presence of considerable antioxidant activity in these edible flowers, although direct numerical comparisons with the present findings are not appropriate because of differences in extraction procedures, assay principles, units of expression, and sample basis.

For *A. officinalis*, Farhat et al. [32] reported total phenolic and flavonoid contents of 16.56 mg GAE/g dry extract and 33.78 mg quercetin equivalents/g dry extract, respectively. Similarly, Shahsavari and Derikvand [33] reported a FRAP value of 1.2 mmol Fe^2+^/L for ethanol extracts of *A. officinalis* and related its antioxidant activity to the presence of phenolic compounds and flavonoids. These compounds act as effective hydrogen- and electron-donating antioxidants through their hydroxyl groups [14], which provides a plausible explanation for, but does not establish the cause of, the antioxidant capacity observed for *A. officinalis* in the present study. Moreover, Memdueva et al. [15] identified a wide range of phenolic acids and flavonoids in *A. officinalis* extracts, including caffeic acid derivatives, rutin, quercetin and kaempferol glycosides, naringenin derivatives, and tilirosides. These phenolic constituents are well recognized for their hydrogen- and electron-donating capacities, and their presence may have contributed to the antioxidant responses observed for *A. officinalis*, although contributions from other extract constituents cannot be ruled out.

Likewise, El-Feky and El-Rashedy [20] reported a TPC value of 81.34 mg GAE/g for methanolic extracts of *V. odorata* flowers. The antioxidant activity of *V. odorata* has been attributed not only to its phenolic and flavonoid constituents but also to other bioactive metabolites, including cyclotides, alkaloids, tannins, saponins, and vitamin C, suggesting that multiple classes of compounds may contribute to its antioxidant potential [21]. This is consistent with the considerable antioxidant activity recorded for *V. odorata* in the present study. Similarly, *V. odorata* has been reported to possess strong in vitro antioxidant activity through multiple free radical scavenging mechanisms, supporting the considerable antioxidant capacity observed both before and after simulated gastrointestinal digestion in the present study [25].

Following the intestinal phase of in vitro digestion, significant reductions (*p* < 0.05) in both TPC and antioxidant activities were observed for all edible flower samples, although the extent of reduction differed among the species and antioxidant assays. Jasmine retained the highest DPPH radical scavenging activity (24.55 µmol TE/g DM), CUPRAC antioxidant capacity (13.71 µmol TE/g DM), and TPC (533.07 mg GAE/100 g) after digestion, whereas marshmallow flower exhibited the highest ABTS antioxidant activity (18.62 µmol TE/g DM). These findings suggest that gastrointestinal digestion may differentially influence the measured antioxidant responses, potentially through changes in compound stability, chemical characteristics, or interactions with the food matrix. The different responses observed for TPC and the individual antioxidant assays further suggest that changes in TPC alone may not fully explain the changes in antioxidant capacity after digestion.

The calculated bioaccessibility indices further demonstrate distinct behaviors among the edible flower species. Marshmallow flower exhibited the highest ABTS (83.23%) and CUPRAC (67.84%) bioaccessibility, indicating that a greater proportion of ABTS-reactive antioxidant compounds became accessible during gastrointestinal digestion. In contrast, jasmine showed the highest bioaccessibility values for DPPH (57.97%) and TPC (60.89%), suggesting that a relatively greater proportion of its DPPH-reactive compounds and total phenolic constituents remained measurable after digestion. Sweet violet generally exhibited intermediate bioaccessibility values for most of the evaluated parameters. The differences among BI values across assays also indicate that gastrointestinal digestion may alter the accessibility or chemical characteristics of antioxidant compounds in an assay-dependent manner.

The differences observed among the antioxidant assays may partly reflect differences in the qualitative and quantitative composition of phenolic constituents, although contributions from non-phenolic compounds cannot be excluded. Both the ABTS and CUPRAC assays can estimate the antioxidant capacity of hydrophilic and lipophilic antioxidants in polar and nonpolar solvent media [33]. In contrast, the FRAP assay is primarily sensitive to hydrophilic antioxidants because of the aqueous affinity of the Fe(TPTZ)^2+^ complex [33]. Likewise, the DPPH assay evaluates free radical scavenging activity and can be applied to both hydrophilic and lipophilic antioxidants when appropriate solvent systems are employed [34]. Consequently, differences in the qualitative composition of phenolic compounds among the edible flowers may have contributed to, but cannot solely account for, the assay-dependent variations observed after digestion. In addition, non-phenolic antioxidant constituents may also contribute to the measured responses, and the relative contribution of individual compound classes may differ among ABTS, DPPH, and CUPRAC assays.

The differences observed among the three edible flower species may partly reflect differences in the qualitative and quantitative composition of phenolic constituents, although contributions from non-phenolic compounds cannot be excluded [28]. Moreover, extraction efficiency is strongly influenced by solvent polarity, extraction time, pH, and temperature, while the differential affinity of individual phenolic compounds for the extraction solvent may further contribute to variations in antioxidant capacity [1,18]. Therefore, the higher antioxidant responses observed in extracts with higher TPC should be interpreted as an association rather than evidence of a direct causal relationship. Collectively, these findings indicate that both the abundance and composition of phenolic compounds may contribute to the antioxidant potential of edible flowers, while other non-phenolic constituents and assay-specific responses should also be considered.

### 3.3. Phenolic Profile

The individual phenolic profiles of the edible flower extracts varied significantly among the three edible flower species and were markedly influenced by simulated gastrointestinal digestion (Table 3). Although all quantified phenolic compounds remained detectable after the IN phase, their concentrations decreased substantially, indicating a lower recovery of the quantified compounds after gastrointestinal digestion. Such reductions are consistent with previous reports demonstrating that gastrointestinal digestion can alter the content and chemical characteristics of phenolic compounds through the combined effects of digestive enzymes, pH fluctuations, and interactions with the food matrix [35]. However, the decrease in the measured concentrations observed in the present study cannot be conclusively attributed to degradation or reduced bioaccessibility, as degradation products and mass balance were not determined. Other factors, including precipitation, adsorption to the matrix or digestion residues, chemical transformation, matrix-related analytical effects, or losses during centrifugation and subsequent sample handling, may also have contributed to the lower concentrations measured after digestion. Therefore, the reduced concentration observed after the intestinal phase should be interpreted as a decrease in the recoverable concentrations of the quantified phenolic compounds rather than definitive evidence of their degradation or limited bioaccessibility. The continued detection of all target compounds after digestion further indicates that none of the quantified compounds was completely absent under the conditions of the present study.

Protocatechuic acid was the predominant phenolic acid in all edible flower extracts, with the highest concentration detected in *V. odorata* (213.51 µg/g), followed by *A. officinalis* (136.35 µg/g) and *J. officinale* (107.35 µg/g). Following intestinal digestion, protocatechuic acid decreased to 78.46, 57.27, and 37.31 µg/g, respectively, corresponding to reductions of approximately 57.99–63.25%. Despite this decline, *V. odorata* retained significantly higher levels than the other species, suggesting greater stability or enhanced release of this compound during digestion.

Catechin also represented one of the major phenolic constituents, reaching 203.53 µg/g in *V. odorata* before digestion. After the intestinal phase, its concentration declined to 93.73 µg/g, whereas *J. officinale* and *A. officinalis* remained 57.26 and 52.47 µg/g, respectively. The marked reduction in catechin concentration after the intestinal phase is consistent with previous reports demonstrating the limited gastrointestinal stability of catechins. Catechins are known to undergo extensive degradation under intestinal conditions, particularly at neutral to slightly alkaline pH, thereby reducing their bioaccessible fraction available for subsequent absorption [36].

The distribution of hydroxybenzoic acid derivatives differed considerably among the edible flower species. *J. officinale* contained the highest concentration of *p*-hydroxybenzoic acid (27.94 µg/g), while syringic acid was most abundant in *A. officinalis* (28.17 µg/g). Both compounds decreased by approximately 51.75–66.73% after digestion, reflecting the limited stability of simple phenolic acids under gastrointestinal conditions. Likewise, ellagic acid, which reached 69.36 µg/g in *V. odorata*, decreased to 22.14 µg/g in the IN phase. The relatively low recovery of ellagic acid is in agreement with previous reports suggesting that ellagic acid is largely associated with the insoluble food matrix, which restricts its release during digestion and consequently lowers its bioaccessible fraction [37].

Among the hydroxycinnamic acid derivatives, *V. odorata* exhibited remarkably higher concentrations of *m*-coumaric acid (82.63 µg/g), *p*-coumaric acid (47.36 µg/g), ferulic acid (73.53 µg/g), chlorogenic acid (114.46 µg/g), and sinapic acid (28.33 µg/g) than the other edible flowers prior to digestion. Although these compounds were significantly reduced during the intestinal phase, *V. odorata* consistently retained the highest residual concentrations, including 19.73 µg/g *m*-coumaric acid, 17.74 µg/g *p*-coumaric acid, 34.35 µg/g ferulic acid, 58.36 µg/g chlorogenic acid, and 9.41 µg/g sinapic acid. In contrast, caffeic acid was more abundant in *J. officinale* (36.78 µg/g) and *A. officinalis* (37.73 µg/g) than in *V. odorata* (8.16 µg/g), with jasmine retaining the highest concentration after digestion (19.33 µg/g), indicating comparatively greater stability of this compound in the jasmine flower matrix.

Among the flavonoids, rutin was one of the most abundant compounds identified in all extracts. *A. officinalis* contained significantly higher rutin concentrations than the other edible flowers, reaching 246.24 µg/g before digestion and remaining the richest source after digestion (62.51 µg/g), despite an approximately 74.61% reduction. Likewise, myricetin was highest in *A. officinalis* (74.62 µg/g) and decreased to 28.73 µg/g after the intestinal phase, while lower concentrations were detected in *J. officinale* and *V. odorata*.

The phenolic profiles obtained in the present study are generally consistent with previous reports describing the phytochemical composition of these edible flowers. For *A. officinalis*, Farhat et al. [32] identified caffeic acid, apigenin, galangin, *p*-coumaric acid, trans-ferulic acid, quercetin, kaempferol, rutin, and gallic acid as the major phenolic constituents, while El-Feky and El-Rashedy [20] reported gentisic acid, apigenin-7-*O*-glucoside, catechin, and rutin as the predominant compounds. Similarly, the present study identified catechin, caffeic acid, rutin, ferulic acid, chlorogenic acid, and several hydroxybenzoic and hydroxycinnamic acid derivatives in *A. officinalis*, confirming that this species is a rich source of bioactive phenolics. These compounds have been widely associated with antioxidant, anti-inflammatory, enzyme inhibitory, and antiproliferative activities through mechanisms involving free radical scavenging, modulation of oxidative stress, regulation of cell-cycle progression, and induction of apoptosis.

Previous studies have also demonstrated considerable variation in the phenolic composition of *J. officinale*, depending on extraction procedures, geographical origin, and analytical methodology. Chen et al. [31] identified epicatechin, ferulic acid, epicatechin gallate, salicylic acid, chlorogenic acid, and epigallocatechin gallate as the major phenolics in jasmine flowers, whereas caffeic acid, syringic acid, quercetin, cinnamic acid, and kaempferol were not detected. In contrast, Yasar et al. [11] reported protocatechuic acid as the predominant phenolic compound together with *p*-hydroxybenzoic acid, syringic acid, rutin, and *o*-coumaric acid, while caffeic acid, ferulic acid, *p*-coumaric acid, *m*-coumaric acid, myricetin, and quercetin was absent. The present findings partially agree with these reports, as protocatechuic acid was also one of the dominant phenolics in *J. officinale*, whereas caffeic acid, ferulic acid, chlorogenic acid, rutin, myricetin, and *p*-coumaric acid were additionally detected. The abundance of these compounds is strongly influenced by plant tissue type, genotype, environmental conditions, harvest stage, and postharvest storage, all of which contribute to the phenolic composition and biological characteristics of plants [38].

The observed differences in individual phenolic composition may also explain the variations in the biological activities of the edible flower extracts. Although *J. officinale* exhibited the highest TPC and antioxidant capacity, *A. officinalis* showed stronger enzyme inhibitory and anti-inflammatory activities. This finding suggests that the biological properties of edible flowers are governed not only by the total amount of phenolics but also by the qualitative composition of individual phenolic compounds and the possible synergistic interactions among diverse phytochemicals.

### 3.4. Amino Acid Profile

The amino acid composition of the edible flower extracts differed significantly among the three edible flower species and was substantially influenced by simulated gastrointestinal digestion (Table 4). Both non-essential and essential amino acids were detected, although their abundance varied considerably depending on the botanical source. While most amino acids decreased following the intestinal phase, several amino acids, particularly in *A. officinalis*, exhibited increased concentrations after digestion. The observed changes in amino acid concentrations following simulated gastrointestinal digestion may reflect alterations in the recoverable total hydrolyzable amino acid fraction under the digestion conditions. Although the increase in certain amino acids is compatible with the release of amino acids from protein or peptide structures during gastrointestinal digestion, this interpretation remains tentative because protein digestibility, amino nitrogen, released peptides, and residual proteolytic activity were not directly measured in the present study. The crude protein contents of the flowers (3.80% for *J. officinale*, 4.74% for *A. officinalis*, and 4.42% for *V. odorata*) provide information on the initial protein content but do not directly indicate protein digestibility or the extent of proteolysis. Therefore, the increase observed for several amino acids, particularly in *A. officinalis*, should be interpreted as changes in the measured amino acid concentrations after digestion rather than as definitive evidence of enhanced proteolytic release [39].

Among the non-essential amino acids, proline was one of the predominant constituents in all edible flowers. The highest concentration was observed in *A. officinalis* before digestion (4.80 mg/g), which further increased to 6.40 mg/g after the intestinal phase. Similarly, aspartic acid and glutamic acid were particularly abundant in *A. officinalis* following digestion, increasing from 3.50 to 4.80 mg/g and from 3.80 to 5.00 mg/g, respectively. Serine, glycine, histidine, leucine, lysine, and valine also showed enhanced concentrations after digestion in marshmallow flowers. These increases indicate a greater measured concentration of these amino acids after the intestinal phase; however, they cannot be conclusively attributed to the liberation of amino acids from endogenous proteins or peptides because protein hydrolysis and peptide release were not directly quantified. In contrast, *J. officinale* and *V. odorata* generally exhibited reductions in the concentrations of these amino acids after digestion, which may reflect differences in the composition of the original matrix, amino acid recovery, chemical transformation, or other digestion-related processes.

Jasmine flower was characterized by comparatively higher concentrations of alanine (3.60 mg/g), tyrosine (5.00 mg/g), phenylalanine (4.00 mg/g), arginine (2.40 mg/g), and lysine (1.00 mg/g) in the undigested samples. Although all of these amino acids decreased after the intestinal phase, jasmine retained significantly higher levels of alanine, tyrosine, arginine, and phenylalanine than the other edible flowers. In particular, histidine and aromatic amino acids such as tryptophan have been reported to contribute to the antioxidant activity of proteins and peptides. Beyond changes in amino acid concentrations, the presence of these residues may have functional relevance because histidine and hydrophobic amino acids, including valine, leucine, phenylalanine, and tryptophan, are recognized contributors to the antioxidant activity of proteins and peptides. Histidine exerts antioxidant effects through hydrogen donation, lipid peroxyl radical trapping, and metal ion chelation via its imidazole group, whereas aromatic amino acids such as phenylalanine and tryptophan can donate hydrogen atoms or electrons to quench free radicals, and arginine serves as a precursor for nitric oxide synthesis and has been linked to various physiological functions, including immune regulation and vascular homeostasis [40,41,42,43].

In contrast, *V. odorata* contained remarkably higher concentrations of several essential amino acids, particularly threonine (5.80 mg/g), methionine (5.50 mg/g), and tryptophan (4.50 mg/g), which remained the highest among all edible flowers even after digestion, despite moderate reductions. The relatively high measured concentrations of these amino acids after digestion indicate that *V. odorata* had comparatively higher recoverable concentrations of these indispensable amino acids under the experimental conditions. Threonine plays a crucial role in maintaining intestinal mucosal integrity through its involvement in mucin synthesis [44], whereas methionine contributes to sulfur homeostasis and one-carbon metabolism as a sulfur-containing methyl donor [45]. In addition, tryptophan functions as a precursor of numerous bioactive metabolites and participates in the regulation of neuronal activity, immune responses, oxidative stress, inflammation, and intestinal homeostasis [46].

Notably, several amino acids exhibited increased concentrations after digestion in *A. officinalis*, whereas most amino acids declined in *J. officinale* and *V. odorata.* Such differences may reflect species-specific differences in protein composition and the behavior of proteins and amino acids under gastrointestinal conditions. Protein digestibility is affected by protein conformation and amino acid side-chain modifications, which can restrict the access of digestive enzymes to cleavage sites and consequently influence the release of total hydrolyzable amino acids during digestion [47]. Protein folding and structural stability, governed by intrinsic interactions and environmental conditions such as pH, further determine the accessibility of proteases to these sites and, consequently, the extent of gastrointestinal hydrolysis [48]. 

Overall, the present findings demonstrate that simulated gastrointestinal digestion modified not only the quantity but also the distribution of individual amino acids in edible flowers. *A. officinalis* exhibited the greatest increases in the measured concentrations of several amino acids following digestion, particularly for acidic and several neutral amino acids, whereas *J. officinale* showed comparatively higher measured concentrations of aromatic amino acids and *V. odorata* remained the richest source of several essential amino acids, including threonine, methionine, and tryptophan. These results indicate that each edible flower possesses a distinct nutritional amino acid profile and that gastrointestinal digestion substantially influences the measured amino acid fraction after digestion.

### 3.5. In Vitro Enzyme Inhibitory Activities

The inhibitory activities of the edible flower extracts against α-glucosidase, α-amylase, LOX, AChE, and BChE are presented in Table 5. Significant differences (*p* < 0.05) were observed among the investigated species for all biological activities. Overall, the inhibitory potential varied according to the target enzyme, suggesting differences in the phytochemical composition and enzyme-binding affinity of the extracts.

Among the tested samples, *A. officinalis* exhibited the strongest inhibitory activity against both carbohydrate-hydrolyzing enzymes, with IC_50_ values of 92.46 µg/mL for α-glucosidase and 78.46 µg/mL for α-amylase. These values were significantly lower than those obtained for *J. officinale* and *V. odorata*, indicating superior inhibitory potency. Although acarbose exhibited the greatest inhibition as the pharmaceutical reference compound, the relatively low IC_50_ values of *A. officinalis* indicate a comparatively strong in vitro inhibitory activity against the carbohydrate-hydrolyzing enzymes evaluated in the present study. However, these findings alone do not demonstrate the ability of the extract to control blood glucose levels or contribute to the dietary management of hyperglycemia in humans.

The inhibitory activity observed in the present study may be associated with the phenolic constituents and other bioactive components of the edible flower extracts. Plant-derived polyphenols are well recognized as natural inhibitors of both α-glucosidase and α-amylase through their ability to interact with digestive enzymes and reduce carbohydrate hydrolysis [21]. Moreover, polyphenols and tannins have been reported to inhibit α-amylase through non-competitive or mixed inhibition mechanisms by interacting with regions outside the catalytic site or with both the free enzyme and the enzyme–substrate complex, thereby decreasing catalytic efficiency [6]. Although enzyme kinetics were not investigated in the present study, similar mechanisms could potentially contribute to the inhibitory effects observed for the edible flower extracts.

Previous studies have also demonstrated that *J. officinale* possesses α-glucosidase inhibitory activity. Chen et al. [31] reported that jasmine flower ranked among the most active extracts in a comparative evaluation of 23 herbal plant species and attributed this activity to its rich phenolic composition. In contrast, the present study revealed that *J. officinale* exhibited the weakest inhibitory activity against both α-glucosidase and α-amylase among the three edible flowers evaluated. Likewise, Litewski et al. [49] reported relatively low α-amylase inhibitory activity for jasmine, although considerable variability has been documented across studies. Such discrepancies are most likely related to differences in extraction solvent, extraction technique, assay conditions, geographical origin, and the plant organ analyzed, all of which are known to influence the recovery of bioactive constituents and their enzyme inhibitory potential [49].

The LOX inhibitory assay revealed a similar pattern, with *A. officinalis* exhibiting the strongest LOX inhibitory activity (IC_50_ = 7.89 µg/mL), followed by *V. odorata* (13.50 µg/mL) and *J. officinale* (18.58 µg/mL) (Table 5). Notably, the IC_50_ value of *A. officinalis* was slightly lower than that of the positive control quercetin (8.19 µg/mL), indicating a comparable LOX inhibitory effect under the experimental conditions. This pronounced LOX inhibitory activity is likely associated with the abundance of phenolic compounds and other bioactive constituents capable of modulating inflammatory responses through the suppression of pro-inflammatory mediators and oxidative stress.

The present findings are supported by previous studies demonstrating the anti-inflammatory potential and LOX-inhibitory activity of these edible flowers. Wang et al. [26] reported that *A. officinalis* flower extract significantly reduced the production of the pro-inflammatory cytokines (IL-6, IL-1β, and TNF-α) while increasing the anti-inflammatory cytokine IL-10 in a rat model. In addition, the extract improved antioxidant status and regulated apoptosis-related signaling pathways, further supporting its anti-inflammatory properties. Similarly, methanolic extracts of *V. odorata* flowers exhibited potent 5-lipoxygenase inhibitory activity, which was primarily attributed to flavonoids including kaempferol 3-O-rutinoside, luteolin 7-O-glucoside, and 5,7-dihydroxy-3,6-dimethoxyflavone [20]. These observations suggest that flavonoids and related phenolic compounds may contribute to the LOX-inhibitory activity of *V. odorata*. Moreover, the strong LOX-inhibitory activity exhibited by *A. officinalis* may be attributed to its rich phytochemical composition. Previous studies have also reported significant anti-inflammatory effects of aqueous *A. officinalis* flower extracts in acute and chronic in vivo inflammation models, indicating that the anti-inflammatory potential of this species is maintained across different experimental systems [23]. Nevertheless, direct comparison between the studies should be interpreted with caution because different biological models and response parameters were employed.

Likewise, the bio-functional properties of *J. officinale* have been associated with its diverse phytochemical composition, including flavonoids (e.g., quercetin), phenolic acids, alkaloids, tannins, and essential oil constituents such as linalool and benzyl acetate [50]. The synergistic interactions among these bioactive compounds are considered to contribute to the antioxidant, anti-inflammatory, and neuroprotective properties of jasmine flowers, which may explain the LOX inhibitory activity observed in the present study.

Distinct inhibitory patterns were observed for the cholinesterase enzymes (Table 5). *A. officinalis* exhibited the strongest AChE inhibitory activity among the edible flower extracts (IC_50_ = 186.37 µg/mL), whereas *J. officinale* showed the greatest BChE inhibition with an IC_50_ value of 195.47 µg/mL. In contrast, *V. odorata* displayed the weakest inhibitory activity against both cholinesterases. Although all extracts were considerably less potent than the reference inhibitor galanthamine hydrobromide (IC_50_ = 3.78 and 3.46 µg/mL for AChE and BChE, respectively), this comparison should be interpreted with caution because the edible flower samples were crude extracts containing complex mixtures of phenolic compounds and other bioactive constituents, whereas galanthamine hydrobromide is a purified single compound; therefore, their IC_50_ values are not directly equivalent in terms of composition or purity. Nevertheless, the observed inhibitory effects suggest that the edible flower extracts contain bioactive constituents that may contribute to cholinesterase inhibition, highlighting the need for further investigation to elucidate the underlying mechanisms and identify the compounds responsible for these activities.

Previous studies support the cholinesterase inhibitory and potential neuroprotective properties of these edible flowers. Saleem et al. [51] reported that *V. odorata* extract inhibited AChE activity while simultaneously enhancing endogenous antioxidant defenses by increasing the activities of superoxide dismutase (SOD), catalase (CAT), and reduced glutathione (GSH), accompanied by a reduction in malondialdehyde (MDA) levels. These findings indicate that the observed biological effects of *V. odorata* may involve both cholinesterase inhibition and modulation of oxidative stress. Likewise, extracts of *J. grandiflorum* flowers have been shown to inhibit AChE, BChE, and monoamine oxidase A (MAO-A), an enzyme involved in the oxidative deamination of monoamine neurotransmitters, with these effects being closely associated with the antioxidant capacity of the extracts [18,52]. Considering the high TPC and antioxidant activity of *J. officinale* observed in the present study, its BChE inhibitory activity may similarly be associated, at least in part, with its phenolic-rich phytochemical profile.

The cholinesterase inhibitory activities observed in the present study are likely associated with the synergistic action of phenolic acids, flavonoids, and other secondary metabolites present in the edible flower extracts. These compounds have been reported to interact with cholinesterases through multiple non-covalent interactions, including hydrogen bonding and π–π stacking, thereby reducing enzyme activity and contributing to neuroprotection [53]. However, the specific compounds responsible for the observed inhibition were not identified in the present study, and the contribution of individual constituents or synergistic interactions cannot therefore be established. Variations in the composition and abundance of phenolic compounds, flavonoids, and other secondary metabolites among the investigated edible flowers may explain the distinct cholinesterase inhibitory profiles observed. Accordingly, *A. officinalis* exhibited the strongest AChE inhibitory activity, whereas *J. officinale* showed the greatest inhibition against BChE. Considering the high total phenolic content and pronounced antioxidant capacity (ABTS, CUPRAC, and DPPH) of *J. officinale* observed in the present study, its relatively strong BChE inhibitory activity may be attributed, at least in part, to its phenolic-rich phytochemical composition. These findings demonstrate that the edible flower extracts exhibited in vitro cholinesterase inhibitory activity; however, they do not by themselves establish neuroprotective or anti-Alzheimer activity.

Overall, the present in vitro results indicate that edible flower extracts possess diverse biological activities beyond their nutritional value. *A. officinalis* exhibited the strongest inhibitory effects against α-glucosidase, α-amylase, AChE, and lipoxygenase, whereas *J. officinale* showed the highest BChE inhibitory activity. These findings provide preliminary evidence supporting the potential of edible flowers as valuable sources of bioactive compounds for further investigation of their functional food and nutraceutical potential. However, since the biological activities were evaluated exclusively under in vitro conditions, further mechanistic, in vivo, and clinical investigations are necessary to verify their bioefficacy and physiological significance.

### 3.6. In Vitro Cytotoxic Activity of Edible Flower Extracts

**Figure 1:**
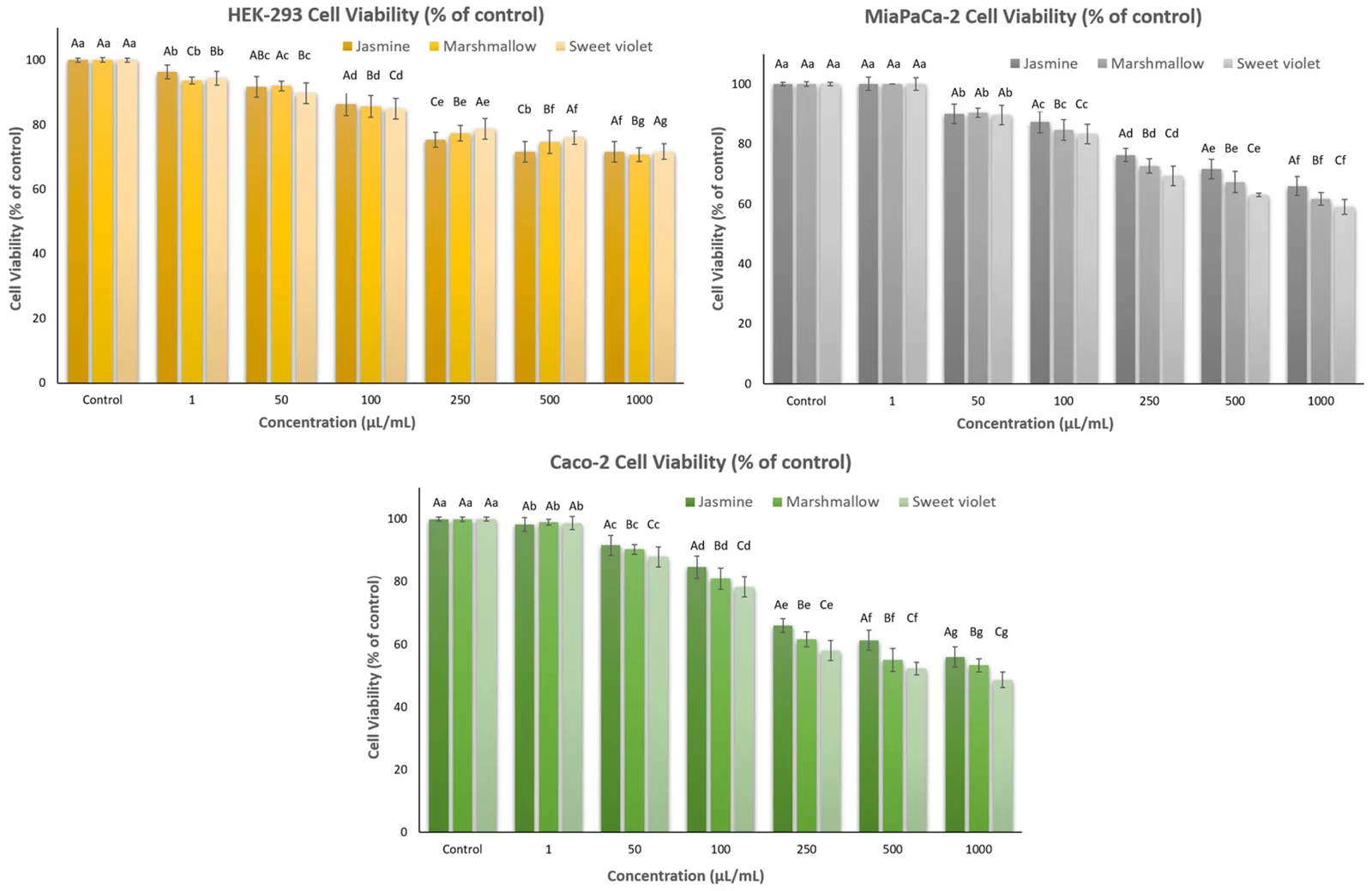
Effects of edible flower extracts on cell viability (%) of HEK-293, Caco-2, and MiaPaCa-2 cells at different concentrations. Values are presented as mean ± standard deviation (*n* = 2). Different uppercase letters (^A–C^) within the same concentration indicate significant differences among edible flower extracts for each cell line, whereas different lowercase letters (^a–g^) within the same row indicate significant differences among concentrations (*p* < 0.05).

In vitro cytotoxic activities of the edible flower extracts against HEK-293, Caco-2, and MiaPaCa-2 cell lines are presented in Figure 1. All extracts reduced cell viability in a concentration-dependent manner (*p* < 0.05), although the magnitude of the response varied according to both the edible flower species and the cell line, indicating differences in the sensitivity of tested cell lines to the extracts. Among the investigated samples, *V. odorata* exhibited greatest reduction in Caco-2 cell viability, with an IC_50_ value of 766.99 µg/mL. In contrast, the IC_50_ values of *J. officinale* and *A. officinalis* against Caco-2 cells were not reached within the tested concentration range and were therefore considered >1000 µg/mL. Similarly, the IC_50_ values for all three extracts against MiaPaCa-2 and HEK-293 cells were not reached at concentrations up to 1000 µg/mL and were therefore considered >1000 µg/mL. At the maximum tested concentration (1000 µg/mL), *V. odorata* reduced the viability of Caco-2 and MiaPaCa-2 cells to 48.67% and 59.00%, respectively, indicating a greater reduction in cell viability for Caco-2 cells than for MiaPaCa-2 cells at the highest concentration tested. Although *V. odorata* exhibited the lowest IC_50_ values against Caco-2 cells among the investigated edible flowers, the IC_50_ values obtained for Caco-2 cells remained well above the concentration range generally considered to represent strong in vitro cytotoxic activity by the National Cancer Institute (NCI) (IC_50_ < 30–40 µg/mL) [3,54], indicating relatively weak in vitro cytotoxic activity under the present experimental conditions. For MiaPaCa-2 and HEK-293 cells, the IC_50_ values were not reached within the tested concentration range and therefore were not extrapolated beyond 1000 µg/mL.

In contrast, all extracts caused less pronounced reductions in cell viability in adenovirus-transformed HEK-293 cells used as a non-malignant comparator, with cell viability remaining above 70% even at the highest extract concentration. Because HEK-293 cells are adenovirus-transformed and do not represent normal human tissue, their relatively high viability should not be interpreted as evidence of a favorable safety profile or selective toxicity toward malignant cells. Because the IC_50_ values were not reached within the tested concentration range, the present data demonstrate only that the extracts caused limited reductions in HEK-293 cell viability under the experimental conditions used. Accordingly, these findings do not establish selective cytotoxic activity, a favorable safety profile, or preferential toxicity toward malignant cells. Further evaluation using additional normal cell lines, broader concentration ranges, appropriate toxicological endpoints, and in vivo studies would be required to assess cellular selectivity and safety [55].

The cytotoxic effects observed in the present study may be associated with the phenolic constituents and other bioactive components of the edible flower extracts. Phenolic compounds are known to affect cancer cell viability through mechanisms involving cell-cycle progression, regulating intracellular oxidative stress, and interfering with signaling pathways involved in tumor growth and angiogenesis [56]. In addition, Kutlu [54] reported that the relatively high TPC of polar plant extracts contributed substantially to their cytotoxic activity. Accordingly, differences in both the composition and abundance of phenolic compounds among the investigated edible flower extracts may contribute to the variations in in vitro cytotoxic activity observed in the present study. However, the specific compounds responsible for the observed effects were not identified in the present study, and a direct causal relationship between TPC and cytotoxic activity cannot be established. More broadly, these findings highlight the need for evidence-based evaluation when interpreting the potential health effects of bioactive-rich plant materials, as the presence of bioactive compounds or their in vitro biological activity alone does not establish health benefits or safety for human consumption [57].

These findings are consistent with those reported by Farhat et al. [56], who demonstrated that aqueous extracts of *A. officinalis* flowers significantly reduced the viability of A549, HCT-116, EB, MCF-7, and HeLa cancer cells in a concentration-dependent manner, whereas no significant cytotoxic effects were observed in normal human dermal fibroblasts. Collectively, these findings provide evidence of in vitro cytotoxic effects of *A. officinalis* flower extracts in the cell models investigated, although differences in extract type, concentration, and cell models may account for variations in cytotoxic potency. These effects were attributed to the presence of flavonoids, including quercetin, rutin, apigenin, and kaempferol, together with phenolic acids such as gallic, caffeic, *p*-coumaric, and ferulic acids, which are known to affect cancer cell viability through mechanisms including apoptosis and disrupting cell-cycle progression.

Similarly, previous investigations have demonstrated the in vitro cytotoxic potential of *V. odorata*. El-Feky and El-Rashedy [20] reported that methanolic flower extracts exhibited marked cytotoxicity against HepG2, Caco-2, and HCT-116 cells, while isolated flavonoids, particularly kaempferol 3-*O*-rutinoside, displayed even greater antiproliferative activity than the crude extract. Likewise, Doğan et al. [16] demonstrated that ethanol extracts of *V. odorata* produced dose-dependent cytotoxic effects against A549 human lung carcinoma cells, with the strongest inhibition observed at 200 µg/mL. Although the IC_50_ values reported in these studies were lower than those obtained in the present work, direct comparisons should be interpreted cautiously because extraction solvents, phytochemical composition, extract standardization, and experimental cell models differed considerably.

Overall, the present MTT-based findings demonstrate that edible flower extracts produced concentration-dependent reductions in cell viability in colorectal and pancreatic cancer cell lines under the tested conditions, while relatively limited effects on cell viability were observed in adenovirus-transformed HEK-293 cells used as a non-malignant comparator. Among the investigated species, *V. odorata* showed the lowest experimentally determined IC_50_ against Caco-2 cells (766.99 µg/mL), whereas *J. officinale* and *A. officinalis* did not reach 50% inhibition within the tested concentration range (>1000 µg/mL). However, the relatively high IC_50_ values and the absence of experimentally determined IC_50_ values for HEK-293 and MiaPaCa-2 cells within the tested concentration range do not establish selective cytotoxicity or food safety. Although these findings should not be extrapolated to in vivo conditions, they provide preliminary evidence that edible flowers warrant further investigation as sources of phytochemicals with potential cytotoxic effects. Further mechanistic investigations together with studies involving additional normal cell models and direct proliferation-related endpoints and in vivo evaluation are required to confirm these biological effects and identify the compounds primarily responsible for the observed cytotoxic responses.

## 4. Conclusions

This study provides a comprehensive comparative evaluation of three edible flowers by integrating nutritional composition, phytochemical characterization, simulated gastrointestinal digestion properties, enzyme inhibitory activities, and MTT-based cytotoxic properties within a single experimental framework. The results demonstrate that each species exhibited a distinct phytochemical profile and differed in the biological activities evaluated. Although *J. officinale* exhibited the highest total phenolic content, antioxidant capacity, and measured phenolic recovery following INFOGEST digestion, *V. odorata* was characterized by a richer profile of several individual phenolic compounds, while *A. officinalis* displayed superior α-glucosidase, α-amylase, and lipoxygenase inhibitory activities, indicating comparatively strong in vitro enzyme inhibitory activities under the experimental conditions used. The amino acid profiles further revealed species-dependent differences in the measured amino acid concentrations after digestion, emphasizing that gastrointestinal digestion influences not only phenolic compounds but also the measured amino acid fraction. Importantly, all edible flower extracts caused greater reductions in viability in Caco-2 and MiaPaCa-2 cells than in HEK-293 cells at the tested concentrations, although these findings do not establish selective anticancer activity or safety. Collectively, these findings indicate that the biological value of edible flowers cannot be evaluated solely on the basis of their initial phytochemical composition, as simulated gastrointestinal digestion may alter the measured concentrations and activities of bioactive constituents. Therefore, incorporating standardized in vitro digestion models can provide additional information on the behavior of edible flower constituents under simulated gastrointestinal conditions. Additionally, *J. officinale*, *A. officinalis*, and *V. odorata* showed distinct nutritional, phytochemical, antioxidant, enzyme inhibitory, and cytotoxic characteristics in the in vitro models employed in this study. These findings support further investigation of edible flowers as potential sources of bioactive compounds, while their suitability for functional food or nutraceutical applications requires further evaluation. However, the present findings are limited to in vitro models and simulated gastrointestinal digestion and cannot be directly extrapolated to in vivo efficacy or safety. Future studies should focus on in vivo validation, identification of digestion-derived metabolites, assessment of safety using additional normal cell models and relevant toxicological approaches, and elucidation of the molecular mechanisms underlying their biological activities to determine their potential relevance to functional food applications.

## Figures and Tables

**Table 1:** Proximate composition and mineral contents of the edible flower samples.

Category	Parameter	Jasmine (*J. officinale* L.)	Marshmallow Flower (*A. officinalis* L.)	Sweet Violet (*V. odorata* L.)
Proximate composition	Dry matter (%)	8.35 ± 0.14 ^c^	11.43 ± 0.04 ^a^	10.57 ± 0.09 ^b^
	Crude protein (%)	3.80 ± 0.12 ^c^	4.74 ± 0.10 ^a^	4.42 ± 0.09 ^b^
	Crude fat (%)	3.34 ± 0.53 ^b^	2.55 ± 0.02 ^c^	4.35 ± 0.03 ^a^
	Crude ash (%)	9.57 ± 0.03 ^b^	11.88 ± 0.04 ^a^	9.59 ± 1.73 ^b^
	Carbohydrate (%)	79.94 ± 0.06 ^a^	69.40 ± 0.09 ^c^	71.07 ± 0.12 ^b^
Mineral composition (mg/100 g dw)	Calcium (Ca)	936.32 ± 11.39 ^c^	1463.23 ± 24.34 ^a^	1268.34 ± 23.62 ^b^
	Potassium (K)	1835.46 ± 117.64 ^b^	1857.46 ± 113.73 ^a^	1637.74 ± 135.71 ^b^
	Magnesium (Mg)	342.45 ± 6.46 ^a^	246.24 ± 0.47 ^c^	255.38 ± 0.78 ^b^
	Phosphorus (P)	194.63 ± 5.35 ^c^	211.58 ± 0.71 ^b^	253.56 ± 1.23 ^a^
	Sodium (Na)	36.35 ± 0.32 ^a^	25.46 ± 0.05 ^c^	33.58 ± 0.06 ^b^
	Iron (Fe)	7.35 ± 0.03 ^c^	11.36 ± 0.02 ^b^	18.39 ± 0.04 ^a^
	Zinc (Zn)	2.45 ± 0.03 ^c^	4.62 ± 0.01 ^a^	3.67 ± 0.01 ^b^
	Copper (Cu)	N.D.	N.D.	0.42 ± 0.01
	Manganese (Mn)	2.35 ± 0.02 ^c^	4.23 ± 0.01 ^b^	5.78 ± 0.02 ^a^

Values are expressed as mean ± standard deviation (*n* = 3). Different lowercase letters (^a–c^) within the same row indicate statistically significant differences among samples (*p* < 0.05), whereas values sharing the same lowercase letter are not significantly different (*p* > 0.05). N.D.: not detected.

**Table 2:** TPC and antioxidant activities of edible flower samples in undigested sample and IN phase.

Name of Analysis	Digestion Phase	Jasmine (*J. officinale* L.)	Marshmallow Flower (*A. officinalis* L.)	Sweet Violet (*V. odorata* L.)
ABTS antioxidant activity (µmol TE/g DM)	Undigested sample	34.21 ± 0.05 ^a^	22.37 ± 0.20 ^c^	28.55 ± 0.07 ^b^
	IN phase	14.40 ± 0.42 ^b^	18.62 ± 0.11 ^a^	6.82 ± 0.09 ^c^
	BI (%)	42.10 ± 1.19 ^b^	83.23 ± 0.29 ^a^	23.90 ± 0.24 ^c^
CUPRAC antioxidant activity (µmol TE/g DM)	Undigested sample	27.43 ± 1.31 ^a^	17.26 ± 0.28 ^c^	22.70 ± 0.09 ^b^
	IN phase	13.71 ± 0.08 ^a^	11.71 ± 0.13 ^b^	7.24 ± 0.06 ^c^
	BI (%)	50.06 ± 2.11 ^a^	67.84 ± 0.90 ^a^	31.87 ± 0.13 ^b^
DPPH radical scavenging activity (µmol TE/g DM)	Undigested sample	42.34 ± 0.18 ^a^	31.36 ± 0.09 ^c^	38.81 ± 0.12 ^b^
	IN phase	24.55 ± 0.74 ^a^	15.20 ± 0.49 ^c^	17.19 ± 0.06 ^b^
	BI (%)	57.97 ± 1.91 ^a^	48.47 ± 0.92 ^c^	44.28 ± 0.10 ^b^
TPC (mg GAE/100 g)	Undigested sample	884.00 ± 20.22 ^a^	663.34 ± 19.12 ^c^	729.40 ± 18.39 ^b^
	IN phase	533.07 ± 9.17 ^a^	329.27 ± 13.57 ^c^	364.22 ± 7.23 ^b^
	BI (%)	60.89 ± 1.77 ^a^	49.64 ± 1.10 ^c^	49.94 ± 0.27 ^b^

DM, dry matter; TE, Trolox equivalents; GAE, gallic acid equivalents; TPC, total phenolic content; BI, bioaccessibility index. Values are presented as mean ± standard deviation (*n* = 3). Different lowercase letters (^a–c^) within the same sample (Undigested sample, IN phase, or Bioaccessibility Index) and within the same analysis indicate statistically significant differences among the edible flower samples (*J. officinale*, *A. officinalis*, and *V. odorata*) (*p* < 0.05).

**Table 3:** The individual phenolic profile of edible flowers in undigested samples and the IN phase of simulated gastrointestinal digestion.

Phenolic Acids (µg/g Extract)	Digestion Status	Jasmine (*J. officinale* L.)	Marshmallow Flower (*A. officinalis* L.)	Sweet Violet (*V. odorata* L.)
Protocatechuic acid	Undigested sample	107.35 ± 0.02 ^c^	136.35 ± 0.03 ^b^	213.51 ± 0.04 ^a^
	IN phase	37.31 ± 0.05 ^c^	57.27 ± 0.01 ^b^	78.46 ± 0.01 ^a^
Catechin	Undigested sample	94.28 ± 0.01 ^c^	157.25 ± 0.05 ^b^	203.53 ± 0.03 ^a^
	IN phase	57.26 ± 0.01 ^b^	52.47 ± 0.01 ^c^	93.73 ± 0.03 ^a^
*p*-Hydroxybenzoic acid	Undigested sample	27.94 ± 0.01 ^a^	16.38 ± 0.01 ^b^	7.36 ± 0.01 ^c^
	IN phase	13.62 ± 0.01 ^a^	6.84 ± 0.01 ^b^	2.46 ± 0.01 ^c^
Syringic acid	Undigested sample	11.46 ± 0.01 ^b^	28.17 ± 0.01 ^a^	5.35 ± 0.01 ^c^
	IN phase	4.72 ± 0.01 ^b^	13.55 ± 0.01 ^a^	1.78 ± 0.01 ^c^
Ellagic acid	Undigested sample	27.46 ± 0.01 ^b^	11.46 ± 0.01 ^c^	69.36 ± 0.01 ^a^
	IN phase	14.13 ± 0.01 ^b^	5.27 ± 0.01 ^c^	22.14 ± 0.01 ^a^
*m*-Coumaric acid	Undigested sample	15.73 ± 0.01 ^b^	11.24 ± 0.01 ^c^	82.63 ± 0.03 ^a^
	IN phase	6.78 ± 0.01 ^b^	6.17 ± 0.01 ^c^	19.73 ± 0.01 ^a^
Caffeic acid	Undigested sample	36.78 ± 0.01 ^b^	37.73 ± 0.01 ^a^	8.16 ± 0.01 ^c^
	IN phase	19.33 ± 0.01 ^a^	9.79 ± 0.01 ^b^	2.42 ± 0.01 ^c^
*p*-Coumaric acid	Undigested sample	19.34 ± 0.01 ^b^	17.46 ± 0.01 ^c^	47.36 ± 0.03 ^a^
	IN phase	18.46 ± 0.01 ^a^	5.83 ± 0.01 ^c^	17.74 ± 0.01 ^b^
Ferulic acid	Undigested sample	42.56 ± 0.02 ^c^	46.83 ± 0.01 ^b^	73.53 ± 0.02 ^a^
	IN phase	13.51 ± 0.01 ^c^	14.62 ± 0.01 ^b^	34.35 ± 0.01 ^a^
Myricetin	Undigested sample	68.68 ± 0.04 ^b^	74.62 ± 0.01 ^a^	31.68 ± 0.01 ^c^
	IN phase	19.28 ± 0.01 ^b^	28.73 ± 0.01 ^a^	18.24 ± 0.01 ^c^
Chlorogenic acid	Undigested sample	89.34 ± 0.02 ^b^	23.62 ± 0.01 ^c^	114.46 ± 0.01 ^a^
	IN phase	33.72 ± 0.01 ^b^	10.39 ± 0.01 ^c^	58.36 ± 0.01 ^a^
Rutin	Undigested sample	129.53 ± 0.02 ^c^	246.24 ± 0.01 ^a^	143.45 ± 0.01 ^b^
	IN phase	41.84 ± 0.02 ^c^	62.51 ± 0.01 ^a^	47.05 ± 0.02 ^b^
Sinapic acid	Undigested sample	14.74 ± 0.01 ^b^	13.64 ± 0.01 ^c^	28.33 ± 0.01 ^a^
	IN phase	4.77 ± 0.01 ^c^	7.22 ± 0.01 ^b^	9.41 ± 0.01 ^a^

Values are presented as mean ± standard deviation (*n* = 3). Different lowercase letters (^a–c^) within the same digestion phase (Undigested sample or IN phase) and within the same analysis indicate statistically significant differences among the edible flower samples (*J. officinale*, *A. officinalis*, and *V. odorata*) (*p* < 0.05).

**Table 4:** Amino acid composition results (mg/g) of jasmine, marshmallow, and sweet violet flowers in the undigested samples and IN phase.

Amino Acid Types (mg/g)	Digestion Status	Jasmine (*J. officinale* L.)	Marshmallow Flower (*A. officinalis* L.)	Sweet Violet (*V. odorata* L.)
L-Aspartic acid	Undigested sample	3.70 ± 0.03 ^a^	3.50 ± 0.03 ^b^	1.60 ± 0.01 ^c^
	IN phase	2.70 ± 0.02 ^b^	4.80 ± 0.04 ^a^	1.20 ± 0.01 ^c^
L-glutamic acid	Undigested sample	3.20 ± 0.04 ^b^	3.80 ± 0.02 ^a^	1.20 ± 0.01 ^c^
	IN phase	2.40 ± 0.03 ^b^	5.00 ± 0.03 ^a^	0.70 ± 0.01 ^c^
L-serine	Undigested sample	2.50 ± 0.03 ^a^	2.20 ± 0.02 ^b^	2.20 ± 0.04 ^b^
	IN phase	1.80 ± 0.02 ^b^	3.00 ± 0.03 ^a^	1.60 ± 0.03 ^c^
Glycine	Undigested sample	1.80 ± 0.03 ^b^	1.80 ± 0.03 ^b^	1.90 ± 0.01 ^a^
	IN phase	1.30 ± 0.02 ^c^	2.40 ± 0.04 ^a^	1.40 ± 0.01 ^b^
L-histidine	Undigested sample	1.10 ± 0.03 ^b^	1.70 ± 0.04 ^a^	1.00 ± 0.01 ^c^
	IN phase	0.80 ± 0.02 ^b^	2.30 ± 0.05 ^a^	0.70 ± 0.01 ^c^
L-Arginine	Undigested sample	2.40 ± 0.03 ^a^	0.80 ± 0.02 ^c^	1.80 ± 0.01 ^b^
	IN phase	1.80 ± 0.02 ^a^	1.10 ± 0.03 ^c^	1.30 ± 0.01 ^b^
L-Threonine	Undigested sample	0.70 ± 0.01 ^b^	N.D.	5.80 ± 0.04 ^a^
	IN phase	0.50 ± 0.01 ^b^	N.D.	4.20 ± 0.03 ^a^
L-Alanine	Undigested sample	3.60 ± 0.04 ^a^	0.80 ± 0.01 ^b^	1.80 ± 0.01 ^b^
	IN phase	2.70 ± 0.03 ^a^	1.10 ± 0.01 ^c^	1.30 ± 0.01 ^b^
L-Proline	Undigested sample	4.50 ± 0.03 ^b^	4.80 ± 0.03 ^a^	3.80 ± 0.04 ^c^
	IN phase	3.30 ± 0.02 ^b^	6.40 ± 0.04 ^a^	2.80 ± 0.03 ^c^
L-Tyrosine	Undigested sample	5.00 ± 0.04 ^a^	1.70 ± 0.02 ^c^	3.20 ± 0.03 ^b^
	IN phase	3.70 ± 0.03 ^a^	2.30 ± 0.03 ^c^	2.40 ± 0.02 ^b^
L-Valine	Undigested sample	1.50 ± 0.01 ^a^	0.90 ± 0.02 ^c^	1.20 ± 0.01 ^b^
	IN phase	1.10 ± 0.01 ^b^	1.20 ± 0.03 ^a^	0.90 ± 0.01 ^c^
L-Methionine	Undigested sample	1.10 ± 0.03 ^b^	0.30 ± 0.01 ^c^	5.50 ± 0.03 ^a^
	IN phase	0.80 ± 0.02 ^b^	0.40 ± 0.01 ^c^	4.10 ± 0.02 ^a^
L-Isoleucine	Undigested sample	0.70 ± 0.01 ^b^	0.40 ± 0.01 ^c^	1.20 ± 0.01 ^a^
	IN phase	0.50 ± 0.01 ^b^	0.50 ± 0.01 ^b^	0.90 ± 0.01 ^a^
L-Leucine	Undigested sample	1.20 ± 0.03 ^b^	1.30 ± 0.03 ^a^	1.00 ± 0.01 ^c^
	IN phase	0.90 ± 0.02 ^b^	1.80 ± 0.04 ^a^	0.70 ± 0.01 ^c^
L-Phenylalanine	Undigested sample	4.00 ± 0.04 ^a^	1.10 ± 0.04 ^c^	3.10 ± 0.01 ^b^
	IN phase	2.90 ± 0.03 ^a^	1.50 ± 0.05 ^c^	2.30 ± 0.01 ^b^
L-Lysine	Undigested sample	1.00 ± 0.01 ^a^	0.70 ± 0.01 ^b^	N.D.
	IN phase	0.70 ± 0.01 ^b^	1.00 ± 0.01 ^a^	N.D.
L-Tryptophan	Undigested sample	0.80 ± 0.01 ^b^	N.D.	4.50 ± 0.04 ^a^
	IN phase	0.60 ± 0.01 ^b^	N.D.	3.30 ± 0.03 ^a^
Total	Undigested sample	38.8	25.8	40.8
	IN phase	28.5	34.8	29.8

Values are presented as mean ± standard deviation (*n* = 3). Different lowercase letters (^a–c^) within the same digestion phase (Undigested sample or IN phase) and within the same analysis indicate statistically significant differences among the edible flower samples (*J. officinale*, *A. officinalis*, and *V. odorata*) (*p* < 0.05).

**Table 5:** In vitro enzyme inhibitory activities of edible flower extracts.

Sample	α-Glucosidase Inhibitory Activity (IC_50_, µg/mL)	α-Amylase Inhibitory Activity (IC_50_, µg/mL)	LOX Inhibitory Activity (IC_50_, µg/mL)	AChE Inhibitory Activity (IC_50_, µg/mL)	BChE Inhibitory Activity (IC_50_, µg/mL)
Jasmine (*J. officinale* L.)	176.35 ± 0.21 ^a^	166.26 ± 0.08 ^a^	18.58 ± 0.40 ^a^	215.56 ± 0.86 ^b^	195.47 ± 0.31 ^c^
Marshmallow flower (*A. officinalis* L.)	92.46 ± 0.04 ^c^	78.46 ± 0.03 ^c^	7.89 ± 0.06 ^d^	186.37 ± 0.57 ^c^	211.82 ± 0.37 ^b^
Sweet violet (*V. odorata* L.)	145.93 ± 0.26 ^b^	102.58 ± 0.07 ^b^	13.50 ± 0.20 ^b^	289.42 ± 1.12 ^a^	308.84 ± 1.35 ^a^
Acarbose	44.73 ± 0.04 ^d^	40.36 ± 0.03 ^d^	-	-	-
Quercetin	-	-	8.19 ± 0.20 ^c^	-	-
Galanthamine hydrobromide	-	-	-	3.78 ± 0.07 ^d^	3.46 ± 0.06 ^d^

Values are presented as mean ± standard deviation (*n* = 3). Different lowercase letters (^a–d^) within the same column indicate statistically significant differences among the tested samples (*p* < 0.05). Acarbose was used as the positive control for α-glucosidase and α-amylase inhibition assays, quercetin for the anti-inflammatory assay, and galanthamine hydrobromide for the AChE (Acetylcholinesterase) and BChE (butyrylcholinesterase) inhibition assays.

## Data Availability

The original contributions presented in this study are included in the article. Further inquiries can be directed to the corresponding author.
